# Development of a Six-Degree-of-Freedom Analog 3D Tactile Probe Based on Non-Contact 2D Sensors

**DOI:** 10.3390/s24092920

**Published:** 2024-05-03

**Authors:** José Antonio Albajez, Jesús Velázquez, Marta Torralba, Lucía C. Díaz-Pérez, José Antonio Yagüe-Fabra, Juan José Aguilar

**Affiliations:** 1 Aragon Institute for Engineering Research (I3A), University of Zaragoza, C/María de Luna 3, 50018 Zaragoza, Spain; 2 Centro Universitario de la Defensa, Ctra. Huesca s/n, 50090 Zaragoza, Spain

**Keywords:** tactile probe, scanning, CMM, calibration, LED–PSD

## Abstract

In this paper, a six-degree-of-freedom analog tactile probe with a new, simple, and robust mechanical design is presented. Its design is based on the use of one elastomeric ring that supports the stylus carrier and allows its movement inside a cubic measuring range of ±3 mm. The position of the probe tip is determined by three low-cost, noncontact, 2D PSD (position-sensitive detector) sensors, facilitating a wider application of this probe to different measuring systems compared to commercial ones. However, several software corrections, regarding the size and orientation of the three LED light beams, must be carried out when using these 2D sensors for this application due to the lack of additional focusing or collimating lenses and the very wide measuring range. The development process, simulation results, correction models, experimental tests, and calibration of this probe are presented. The results demonstrate high repeatability along the X-, Y-, and Z-axes (2.0 µm, 2.0 µm, and 2.1 µm, respectively) and overall accuracies of 6.7 µm, 7.0 µm, and 8.0 µm, respectively, which could be minimized by more complex correction models.

## 1. Introduction

The current scenario of tighter and tighter quality requirements for products and pressure to shorten production cycles has driven manufacturers to seek accurate but faster methods for completing inspection tasks. Analog tactile probes, also known as scanning or continuous contact probes, are one of the most popular solutions for users of coordinate-measuring machines (CMMs). They perform scanning probing via a tip continuously in contact with the surface under examination. Hence, these probes are able not only to measure complex free-form surfaces but also to collect large numbers of points and execute fast measurements with no restrictions on the reflectiveness of the surface, in contrast to optical measuring systems [1].

While touch-trigger probes were conventionally regarded as the most accurate option due to their point-by-point measurement, this assertion is not as clear nowadays [2]. Tactile probes show certain limitations: most of the commercial probes share some features, such as a measurement precision of around 1 µm or measuring ranges smaller than ±1.5 mm, albeit with (stand-off) ranges of up to 3 mm. Despite having this measuring range, they achieve their optimal performance only when operating with very low deflections. Moreover, these probes are primarily designed for CMM applications [3,4], resulting in a lack of specific calibration procedures out of the machine [5,6]. Furthermore, regarding their internal architecture, these probes often constrain three of their degrees of freedom while measuring the displacement on the other three. 

Due to the need for better resolution and accuracy, in recent years, research on micro-manufacturing and nanotechnology has led to a notable development of ultra-precision devices with diverse architectures [7,8]. These devices, whether scanning probes [9] or touch-trigger probes [10], are designed to move with six degrees of freedom but typically with ranges of around 0.1 mm. Alternatively, silicon microprobes with integrated piezoresistive strain gauges, which measure the deflection of a cantilever, have been successfully developed for industrial applications of roughness and profile measurements [11,12]. However, these new designs are not suited for conventional general-purpose industrial applications. Whether for large-range CMMs, for use on machine tools, or for high-speed applications, the requirements for the control systems would be very tight [13,14]. 

In this paper, a six-degree-of-freedom analog tactile probe with a simple and robust mechanical design is presented. The main feature of this probe is its wide measuring range (±3 mm), not only a wide travel range, allowing for its use without tight control requirements or restrictions to small deflections inside a small measuring range. This characteristic is especially important in applications such as high-range CMMs, whose errors due to elastic deformations are bigger than in conventional CMMs, high-speed applications and scanning parts with abrupt changes on their surfaces, mainly in closed-loop scanning, and easing the adaptation to commercial control, even to machine tools (MTs). Other important features of this probe are its low measuring force (<1 N), resolution, and uncertainty, all of which are similar to existing commercial systems but with a lower cost, which is one of the main design constraints chosen for its development. As will be described in the following section, the proposed design is so robust that its performance does not depend on high-quality components or high manufacturing tolerances: the final accuracy is guaranteed thanks to calibration and error-correction procedures.

The size and weight of the system are also affected by the proposed applications for CMMs and MTs, and it could be used by design as a self-centering [15] or measuring probe. Thus, the developed probe could be integrated as an on-machine measuring system, which may be particularly useful for self-optimizing production systems, e.g., machine tool verification processes [16,17,18], or for surface form inspection [19]. 

A specific calibration procedure for passive-contact analog probes is also described to effectively separate machine and probe errors. Furthermore, the development process, correction models, experimental tests, and calibration of this probe are presented in this paper.

## 2. Probe Description

**Figure 1:**
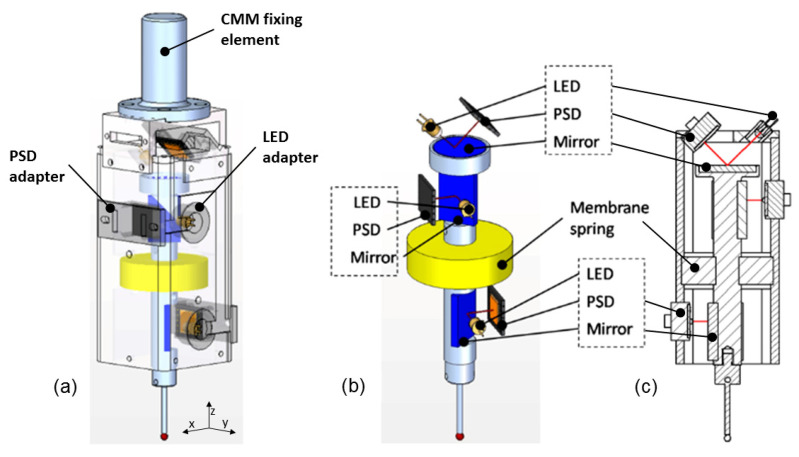
Analog tactile probe developed: complete view (**a**), internal body with sensor system (**b**), and 2D cross-section (**c**).

The developed passive (without internal actuators) tactile probe should meet the requirements of a ±3 mm measuring range on the X-, Y-, and Z-axes and a probing force of up to 1 N. Additionally, its design supports the stylus carrier and allows its six degrees of freedom (DoFs). The final scheme of the probe is presented in Figure 1. As is shown, the sensor system consists of three LED–PSD pairs and mirrors, and a membrane spring to suspend the probe, which are all protected by aluminum housing. The size of the body is 115 × 45 × 45 mm, which is compact enough to be supported by any commercial CMM or MT head.

**Figure 2:**
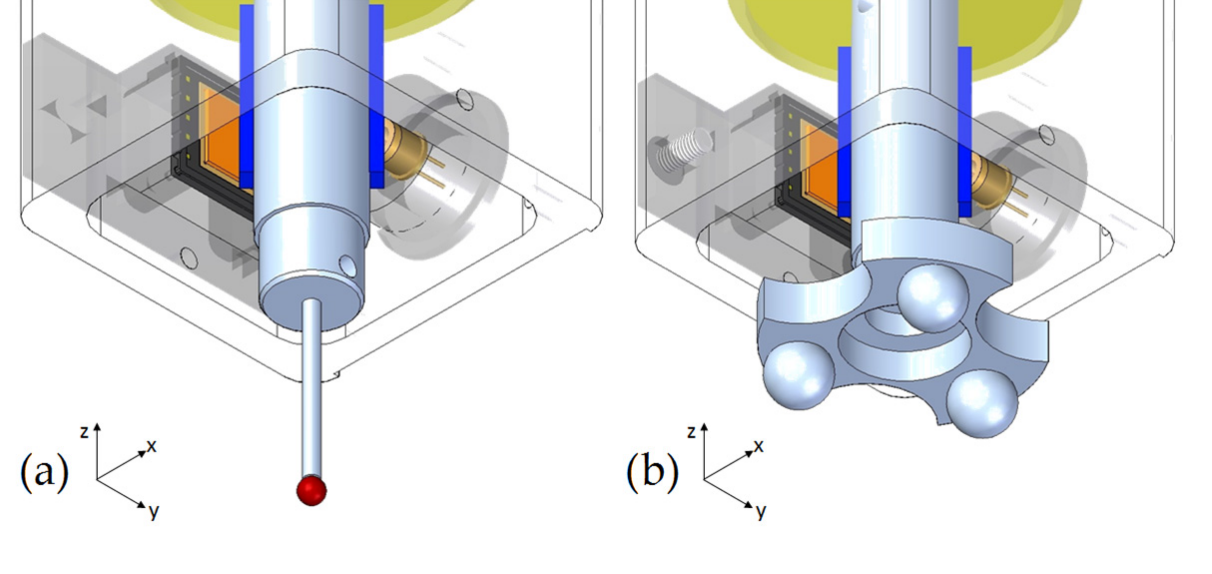
Analog tactile probe heads: scanning probe tip (**a**) and self-centering tip (**b**).

To address a wide range of applications, the probe can be installed with two different types of tips (see Figure 2) to work as a scanning probe (for coordinate-measuring tasks) or as a self-centering probe (for machine tool verification tasks) [15]. The self-centering tip consists of a nest of three spheres that allows for measuring the center of reference spheres of a calibrated ball bar in one single movement. This is very time-efficient compared to an MT with a traditional touch-trigger probe, which needs to sequentially capture several points on each sphere. This is part of a technique whereby the geometrical errors of machine tools are evaluated by comparing the calibrated nominal ball positions with the actual ones reached by the machine and measured by the probe.

Of the possible alternatives analyzed to measure six degrees of freedom with high precision (piezoelectric sensors, capacitive sensors, optical encoders, laser systems, etc.) [20,21], three 2D non-contact sensors were ultimately used. Three light-emitting diodes (LEDs) generate three light beams that are reflected by mirrors. The reflected beams are received by three 2D position-sensitive detectors (PSDs), providing a total of six electronic signals. These six data points, combined through the mathematical model, allow the determination of the six DoFs of the suspended body; therefore, the stylus tip position can be calculated. Despite the nonlinearities in the PSD sensors, LED–PSD was chosen due to its low cost, small size, and easy integration. This is ideal for this application, where CMMs or machine tools could be limited by the weight of the probe. Nevertheless, the extensive work carried out to correct this lack of linearity is also explained in this paper. The selected PSD sensors were pin-cushion-type model Hamamatsu S5991 with an active area of 9 × 9 mm^2^. The LED used (Hamamatsu L2791-02) was selected according to its directivity and peak emission wavelength, which is in the reception spectrum range of the PSD.

**Figure 3:**
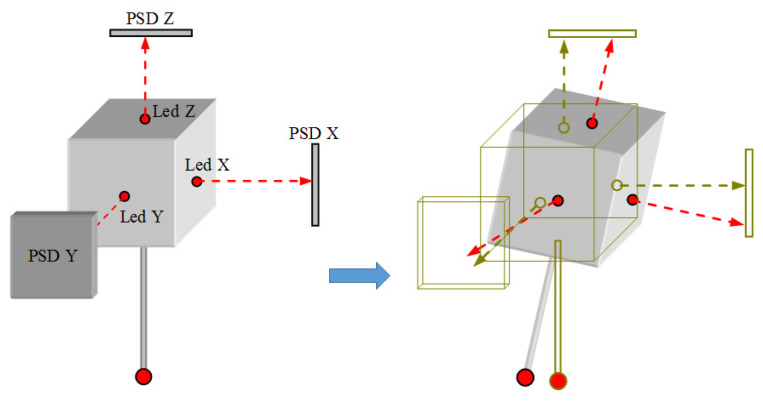
Six degrees of freedom measuring concept of the suspended probe body (the arrows show the light beams emitted by the LEDs).

The main idea to fully understand how it is possible to measure the six degrees of freedom of the suspended body involves considering a simplified view of the body as a free cube in space. According to Figure 3, if the LEDs could be mounted on the cube surfaces, the three emitting rays could be considered as the three axes of a local coordinate system. By measuring the displacement of the rays over the 2D PSD surface when the cube moves, it is possible to reconstruct the movement of this virtual coordinate system and, hence, the relative movement between the final and initial cube positions.

Several considerations must be taken into account in order to install the sensors in an adequate relative position inside the probe. An initial design was defined by locating the PSD sensors on the frame of the probe and the LEDs on its holder. This scheme is particularly simple to model due to the alignment between the LED beams and the Cartesian coordinates (equivalent to Figure 3). Nevertheless, to avoid motion limitations due to wire connections and facilitate the adjustment of the sensor measurement range, three 21 mm × 15 mm first-surface mirrors (Edmund Optics) were attached to the final proposed design (see Figure 1). The size and weight of the probe should be considered by the design; thus, compactness and lightness are desirable features. Consequently, the whole probe configuration was established as follows: the body of the probe is the moving part of the complete system, and it is made of steel. The three mirrors are attached to it considering the X-, Y-, and Z-axes. At the end of the body, a threaded hole allows the interchangeability of the different heads (see Figure 2). The body is connected to the fixed part or frame of the probe by one membrane spring with a 12 mm thickness, made from an elastomeric material (Sylomer G12). Its design and positioning ensure a low probing force (<1 N) and the six degrees of freedom of the probe. Some commercial probes have a serial-kinematics structure with three translational stacked axes. Moreover, it has fewer moving parts, which allows a very compact and simple mechanical design. An additional benefit of this solution is its robustness. This is especially useful for machine tools due to its harsher working conditions and greater probability of impacts. The frame of the probe made of aluminum encompasses the three LED–PSD pairs and serves as structural support. At the top of this housing, the whole system can be installed in CMMs or MTs. All the wiring connections are also run through this part.

**Figure 4:**
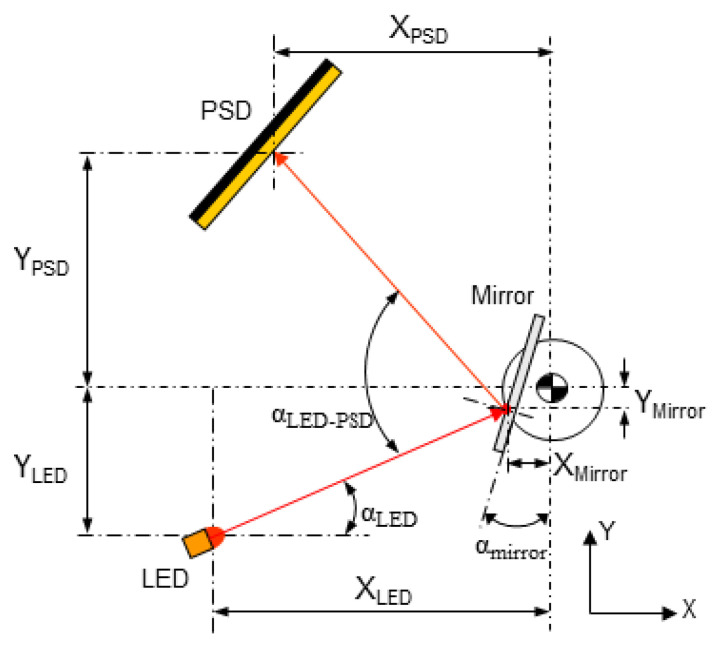
Geometry simulation of relative position and orientation between the LED, mirror, and PSD.

In addition, the design of the system is mainly affected by some influence factors that decrease the measurement accuracy and must be taken into account. For the selected 2D sensors, several error sources must be considered by design, particularly the distance between LED and PSD, alignment, and light beam orientation. Considering this, previous studies carried out by the authors characterized the performance of 1D PSD sensors [22]. Through the application of a similar experimental test procedure, the repeatability of the 2D LED–PSD system was evaluated in this work. The obtained repeatability for the X- and Y-axes of the PSD was 1.1 μm and 1.0 μm, respectively. Secondly, the relative positioning and orientation between components of the sensor system (i.e., LED, mirror, and PSD) were analyzed through the simulation of different relative orientations between the LED and PSD (see Figure 4) and via experimental tests, before reaching the final configuration.

## 3. Geometrical Model

**Figure 5:**
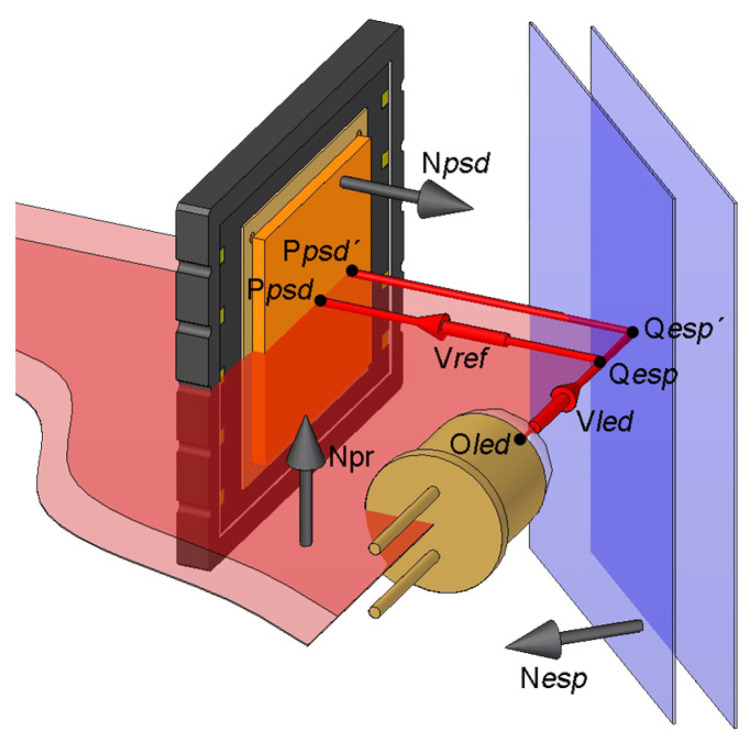
Plane formed by the emitted and reflected light beams when the respective mirror moves.

The geometrical model used to calculate the position of the probe tip was based on the one presented by Van Vliet in [23,24]. This model uses a separate calculation of the rotation and translation parts of the movement, taking advantage of the fact that the light beams leaving the LED and reaching the PSD are always part of the same plane, whenever the mirror only suffers pure translational movements. This is represented in Figure 5. The origin of the LED light source is located at O_led_. Q_esp_ is the point of the mirror where the beam is reflected and P_psd_ is the point where the light beam reaches the PSD. The N_pr_ plane (defined by its normal vector) formed by the emitted light vector (V_led_) and the reflected one (V_ref_) remains invariant at all positions of the probe when the mirror translates. In other words, changes in the reflected point (Q_esp_ → Q_esp_’) or in the measured position in the PSD (P_psd_ → P_psd_’) by translating the mirror do not affect the direction of the reflected light beam (V_ref_).

This geometrical model does not take into account the corrections that have to be carried out concerning the size and shape of the light beam. The model used was applied under the supposition of using one-dimensional light beams (laser diodes in combination with optical elements, as used in [24]). However, the light beams emitted by the LEDs used in this work have a conical profile. The study and correction of this influence is presented below. In addition, the importance of working with light beams perpendicularly oriented with respect to the PSD surface is also demonstrated.

### 3.1. Correction of the Nonlinearity of the Measuring System

**Figure 6:**
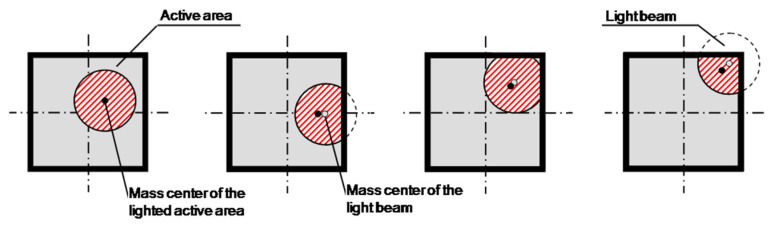
Effect of the light beam size (red area) on the position calculation.

**Figure 7:**
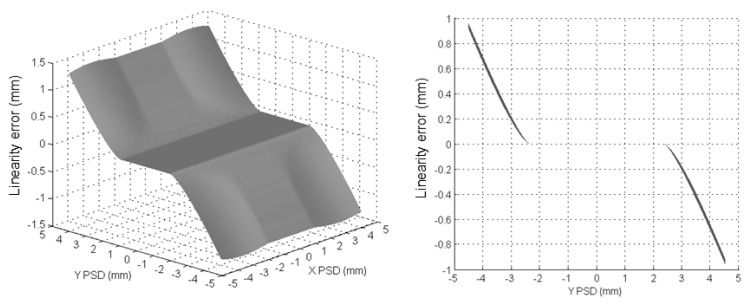
Linearity error for the Y-axis of the PSD obtained via simulation.

The influence of the conical profile of the light beam is as shown in Figure 6. When the light beam reached the PSD surface close to its edges, part of the beam fell outside of the sensitive area, obtaining a calculated mass center of the light beam that was different from the real one. The longer the distance between the LED and PSD, and the closer to the edges of the PSD, the more significant this effect became. Simulations were carried out in order to quantify this effect. According to the directivity of the light source and the probe design, an emitting angle of ±3.5° for the LED light beam and a total distance of 35 mm between the LED and PSD were taken for simulation. Figure 7 shows the linearity errors obtained for the Y-axis of the PSD.

**Figure 8:**
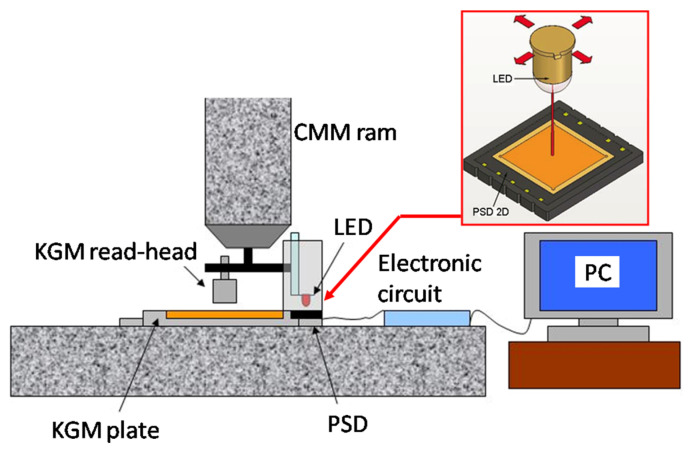
Experimental setup for linearity analysis.

In addition to this, the PSD itself showed nonlinearities in its performance. Experimental tests were carried out to corroborate the simulation results and correct both effects influencing the linearity of the system. A cross-grid encoder (KGM Heidenhain) was used as a reference standard to analyze and correct the linearity of the LED–PSD measuring system. For the experimental setup (see Figure 8), the PSD was fixed on the base of the KGM, on a CMM table. The LED and the KGM reading head were mounted together on the CMM arm. The LED and PSD were separated by 35 mm, as in the probe arrangement. A meticulous alignment process was required to assure the right position between the LED and PSD, as well as the KGM head and grid. A box was used to cover the setup in order to protect it from environmental error sources (ambient light and temperature changes).

The accomplished tests implied the simultaneous acquisition and comparison of the signals provided by both the KGM and the PSD all over its sensitive surface. By using the CMM arm as a motion system, measurements were taken in the PSD active area of 9 mm × 9 mm in a grid with a 0.1 mm pace. Approximately 8500 different points were finally stored in total. It must be noted that there was a horizontal offset (about 70 mm) between the LED and the KGM head and, therefore, the influence of any yaw movement of the X- or Y-axes of the CMM in the final error. This influence was calculated, obtaining errors below ±0.4 µm, which was smaller than the repeatability of the PSD itself (±2 μm); thus, its influence can be neglected.

**Figure 9:**
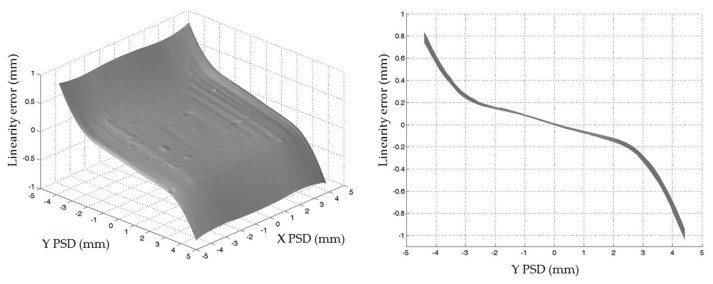
Linearity error for the Y-axis of the PSD obtained via experimentation.

**Figure 10:**
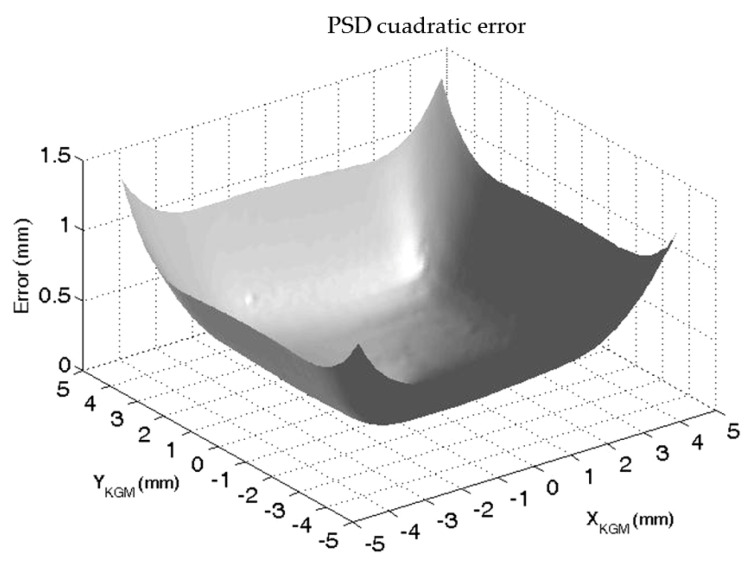
Quadratic error for the XY plane of the PSD obtained via experimentation.

As shown in Figure 9, the experimental results corroborated the results obtained via simulation (Figure 6) and included the intrinsic nonlinearity of the PSD itself. Thus, the obtained error profile was due to not only the lack of linearity of the sensor (around ±150 μm in the ±2 mm central area) but also the size of the light beam as a consequence of the LED–PSD distance. The absolute value of the error rapidly increased when the beam fell out of the central photosensitive area, which was ±2 mm both on the X- and Y-axes. Figure 10 shows the results obtained for the influences on both the X- and Y-axes of the PSD when they were combined, and the quadratic error is represented.

A theoretical model was initially proposed to predict this error quantitatively. However, it was unfeasible due to the correlations between the influence variables in the measurement result. For this reason, an experimental correction model was developed. In view of the form and symmetry of the distortion obtained in Figure 9, which is similar to the distortion shown in optical systems, it was initially decided to apply a model based on Faugeras and Toscani’s method [25], but in patches. These nonlinearities were corrected by dividing the PSD into square areas (0.6 × 0.6 mm in size) and applying different, fully second-order correction equations for each of them, which were as follows:

(1) $$\begin{array}{l} X_{KGM}=a\cdot X_{PSD}+b\cdot Y_{PSD}+c\cdot X_{PSD}\cdot Y_{PSD}+d\cdot X_{PSD}^{2}+e\cdot Y_{PSD}^{2}\\ +f\cdot X_{PSD}^{2}\cdot Y_{PSD}^{2}+g\cdot X_{PSD}\cdot Y_{PSD}^{2}+h\cdot X_{PSD}^{2}\cdot Y_{PSD}+i \end{array}$$

(2) $$\begin{array}{l} Y_{KGM}=j\cdot X_{PSD}+k\cdot Y_{PSD}+l\cdot X_{PSD}\cdot Y_{PSD}+m\cdot X_{PSD}^{2}+n\cdot Y_{PSD}^{2}\\ +o\cdot X_{PSD}^{2}\cdot Y_{PSD}^{2}+p\cdot X_{PSD}\cdot Y_{PSD}^{2}+q\cdot X_{PSD}^{2}\cdot Y_{PSD}+r \end{array}$$

**Figure 11:**
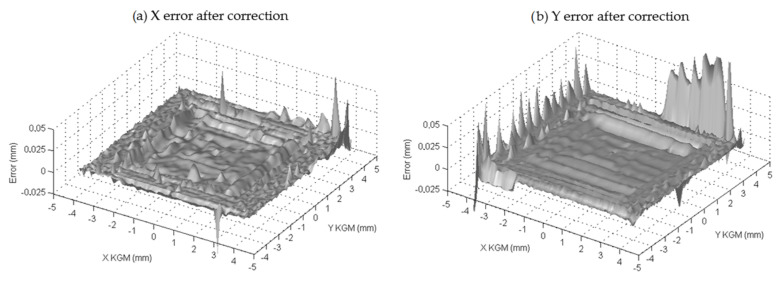
X-error (**a**) and Y-error (**b**) of the PSD after correction, considering KGM as a reference standard.

The correction factors (a, b, c, …, r) were obtained from the experimental tests, taking the KGM as the reference standard. The final maximum errors shown by the PSD over its area after correction were smaller than ±2.5 μm in 85% of the sensitive area around the PSD center (see Figure 11). Here, 15% of the area closest to the edges of the PSD, where the nonlinearities were even more present, showed larger maximum errors after correction, but this could be due to noisy data; border areas, especially corners, received a much lower light intensity (the center of the LED beam is clearly out of the sensing area). Hence, in order to avoid derived measurement inaccuracies from this issue, the analog probe only uses 80% of the sensor measurement range. Other correction models could be applied in the future to this kind of sensor arrangement. For example, three different techniques were considered in [22]: calculating a simple polynomial for each of the dividing parts, using a single polynomial to model the whole error of the PSD along its area, and using a neural network model strategy. Other relevant examples can be seen in [26,27].

### 3.2. Simulation of the Light Beam Orientation on the PSD

**Figure 12:**
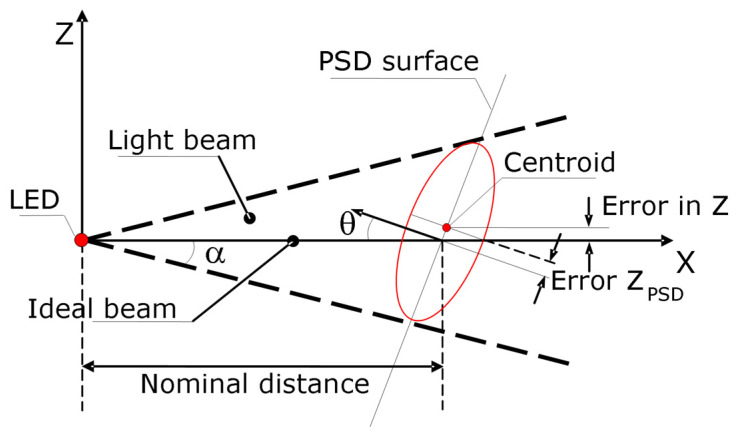
Simplified scheme of the measuring error due to the incident angle of the cone of light on the PSD (red ellipse shows the intersection between the light cone and the PSD surface).

Another important influence that was analyzed is the lack of squareness between the light beam and the PSD active area. In other words, if the cone of light of the LED is not perpendicularly oriented with respect to the PSD, the centroid of the lightened area and the point where the cone axis falls on the PSD surface are not coincident. This is illustrated in Figure 12, where the situation for a simpler case is schematized. In this example, the system has lost its perpendicularity only with respect to one of the axes by rotating a *θ* angle around the Y-axis, showing a Z_PSD_ error. In a general case, a Y_PSD_ error would also appear. 

**Figure 13:**
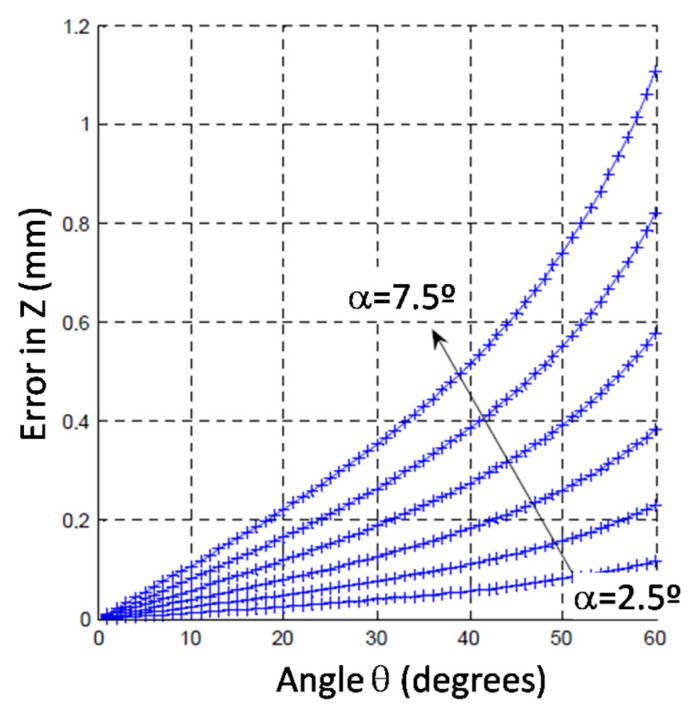
Simulation results of the error due to light beam orientation on the Z-axis for different θ and α angles.

Simulation results carried out to characterize these errors are shown in Figure 13. These results showed that the larger the *θ* angle (meaning the lack of perpendicularity of the light beam) and α angle (meaning the opening radiation angle of the cone light), the greater the errors obtained. Figure 13 shows how the error along the Z-axis was increased for a distance between the LED and PSD of 35 mm and with θ and α angle variation ranges of 0–60° and 2.5–7.5°, respectively. These considerations were taken into account in the design process, and a nominal θ = 0° and an LED with an emission angle α = ±3.5° were used. Hence, the value of θ changed when the probe was not at its initial nominal position. Nevertheless, the influence of those changes was calculated to be corrected for the geometry of the probe and its measuring range, with an average correction factor of θ at the initial probe nominal position; if the value of θ was not very high (according to the probe design), its effect was quite linear, as can be seen in Figure 13.

## 4. Calibration Setup

The calibration of the probe was performed using an out-of-machine calibration technique with sub-micrometer repeatability for passive contact analog probes developed by the authors and described in [28]. This technique is effective for determining the static errors of passive analog probes independently on the errors of the machine or the control where the probe is connected. An alternative approach for touch-trigger probes can be seen in [29]. 

**Figure 14:**
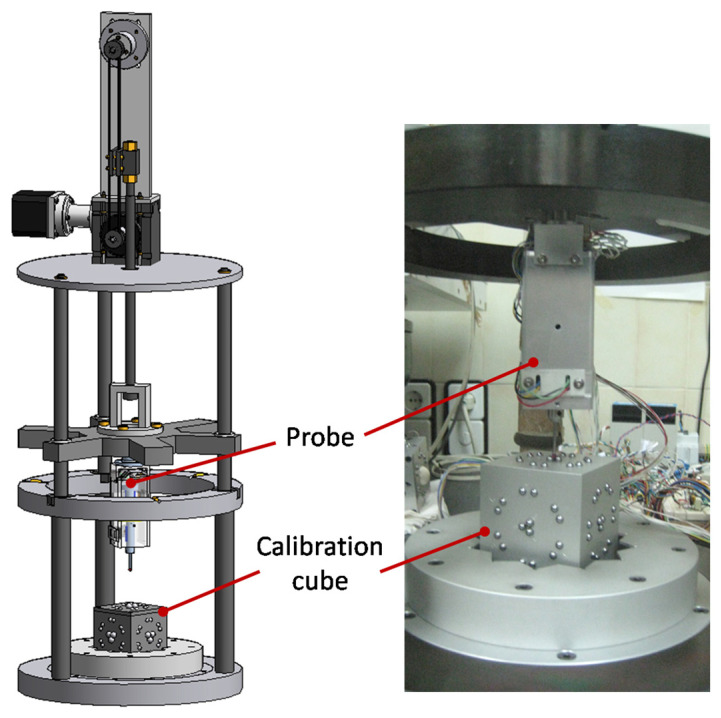
Calibration setup and calibration reference cube for the scanning probe tip.

As shown in Figure 14, the calibration scheme was composed of a standard cube based on spheres for kinematic couplings over a reference base plate and a specific setup to repeatedly place the probe with respect to the cube.

The standard artefact developed consisted of a cube with groups of spheres fixed to its surfaces (Figure 14). Several sets of locating elements (six pairs of 5 mm spheres) were placed on every face in order to precisely position the device on a reference base plate (resting on three cylinders by a magnetically preloaded kinematic coupling). The locating elements and the spheres to be probed had different relative positions and orientations on each face. In total, 36 positions (6 positions on 6 faces, denominated as 1A to 6F) were obtained, covering a range up to ±3 mm in the X, Y, and Z directions. This artefact was calibrated by measuring the positions of its three-sphere nest with a CMM, obtaining the local uncertainties included in Table 1 and the corresponding global uncertainties shown in Table 2.

In addition to the standard artefact, a specific device was developed to locate the probe with respect to the calibration cube with enough repeatability, as described in [28]. The probe was mounted on a moving part that moved up and down thanks to a motion system (Figure 14). When the probe was up, the position of the cube could be manually changed. When the probe was down, it rested on a very repeatable positioning system (±0.1 µm), where the measuring was carried out. It was again based on a passive kinematic coupling seat formed by a three-sphere nest on top of every cube face. This calibration setup did not constrain different orientations.

## 5. Experimental Results

The repeatability and accuracy of the probe were analyzed using the previously explained out-of-machine technique. The experimental tests took place in a metrology laboratory with standard controlled conditions, including a temperature of 20 ± 1 °C and 50–70% humidity. The probe was also isolated using a methacrylate cover around the test setup in order to minimize the influence of possible temperature changes inside the range of control. Repeated measurement results of the probe were compared to the reference values of the standard (reference coordinates of the cube characterized by a CMM). This information was used to fine-tune (mathematical optimization) some of the geometrical values of the probe and absorb differences between the ideal geometry (CAD file) and the real geometry after manufacturing and assembling all the components. The final results obtained using the analog scanning probe are summarized in Table 3, which show the repeatability values for the X-, Y-, and Z-axes after measuring the reference-cube-locating elements in five series, shown as the maximum standard deviation obtained from all those measurements. The systematic error range is also presented, calculated as the deviation of the measurement result from the cube reference.

In view of these results, it was possible to affirm that the probe presented high repeatability along the three axes. The accuracy was also evaluated. The described calibration setup was used to calibrate the probe and estimate random errors. The 36 positions inside a range of ±3 mm in each axis were measured in 10 sets by the probe. Therefore, the uncertainty values, U_i_, obtained for the X-, Y-, and Z-axes in each i position (i = 1A, …, 6F) were calculated using Equation (3):

(3) $$U_{i}(k,n_{i})=k\sqrt{{\left(\frac{U_{cube}}{k_{cube}}\right)}^{2}+s_{ci}^{2}\left(\frac{1}{n_{ci}}+\frac{1}{n_{i}}\right)+{\left(\frac{\Delta x_{i}}{k}\right)}^{2}}$$

where k is the coverage factor (k = 2 for a confidence interval of 95.45%), U_cube_ and k_cube_ are the uncertainty and coverage factor of the standard artefact, respectively (see Table 2), *s*_ci_ is the standard deviation of the calibration (for the i position), n_ci_ and n_i_ are the number of repeated measurements (for the i position) during probe calibration and probe measurement, respectively, and ∆x_i_ is the uncorrected measurement bias (for the i position). The influence of the temperature during the tests was minimized by isolating the calibration setup, as in the previous test.

The results after the compensation for local uncertainty in all the i positions (k = 2, n_i_ = 1) are shown in Table 4. As previously stated, the biggest contributor was uncorrected bias (∆x_i_). Applying more complex optimization procedures (as explained in Section 3.1 and Section 3.2) could reduce these systematic errors and minimize the uncertainty value (U_i_), which is a focus for future work.

**Figure 15:**
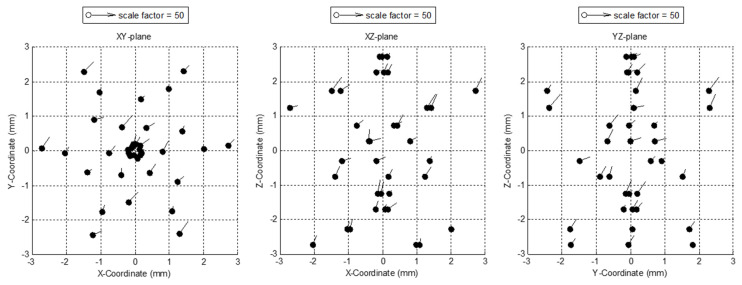
Error mapping results of the analogue tactile probe over the ±3 mm measuring range.

The Table 4 results and the corresponding error mapping in the range of ±3 mm represented on the XY-, XZ-, and YZ-planes are included in Figure 15. The final results for global uncertainty that considers the maximum value from each X-, Y-, and Z-axis between all positions were 6.7 µm, 7.0 µm, and 8.0 µm, respectively, as summarized in Table 5. Nevertheless, it should be noted that the option exists to improve these results by reducing the working range to a more conventional value, as commercial probes do.

## 6. Conclusions

The probe presented in this paper achieved the intended objectives of a high range and low cost through its original design, which constitutes a primary contribution of this work. Two key points supported this claim. Firstly, the probe only had a few moving parts due to the use of an elastomeric membrane spring, resulting in a robust, compact, and mechanically simple design that did not require tight manufacturing tolerances. Secondly, the number and characteristics of electro-optical components were quite limited. Nevertheless, despite the lower cost of this design, the probe was characterized by high repeatability along the three axes, and the uncertainties across its measuring range were similar to the ones demonstrated by the commercial probes working with their recommended low deflections. The uncertainty results showed values below 8 µm for all three axes in all the positions analyzed in a full ±3 mm measuring range, much greater than the one used in existing probes. In this case, it is clear that there was a tradeoff between hardware investment (the quality of components and use of additional optical elements) and software correction strategies, with the latter approach being the one chosen to guarantee low cost. Specifically, two problems were addressed through this strategy: the nonlinearity due to the light beam size was experimentally characterized, and the shape of the light spot was theoretically modeled. However, these results could be further improved by minimizing the uncorrected bias considered in the uncertainty budget. Hence, future research will be focused on considering other correction models that could be applied to the LED–PSD sensor arrangement used, such as using a neural-network model strategy. 

After optimizing the performance of the probe along its high range, it could be very suitable for use in large CMMs, machine tools, or high-speed scanning applications, where the ±3 mm range allows for absorbing trajectory errors or even small collisions. Moreover, it should be noted that the probe could be used as a self-centering probe for machine tool verification, simply by changing the probe tip. 

Finally, the performed design concepts, model corrections, and experimental tests can be adapted for other micro- and nano-technology probe applications.

## Figures and Tables

**Table 1:** Uncertainty results of the 36 positions of the calibration cube.

Sphere Cube Position	U_X_ (k = 2, n_i_ = 1) (µm)	U_Y_ (k = 2, n_i_ = 1) (µm)	U_Z_ (k = 2, n_i_ = 1) (µm)	Sphere Cube Position	U_X_ (k = 2, n_i_ = 1) (µm)	U_Y_ (k = 2, n_i_ = 1) (µm)	U_Z_ (k = 2, n_i_ = 1) (µm)
1A	0.3	0.1	0.8	4A	0.4	0.1	0.9
1B	0.4	0.1	0.8	4B	0.4	0.3	1.0
1C	0.4	0.3	0.8	4C	0.4	0.2	0.9
1D	0.4	0.1	0.8	4D	0.3	0.3	0.9
1E	0.4	0.2	0.8	4E	0.4	0.3	0.9
1F	0.3	0.2	0.8	4F	0.4	0.2	0.9
2A	0.5	0.4	0.8	5A	0.3	0.3	0.9
2B	0.3	0.1	0.8	5B	0.4	0.3	0.9
2C	0.6	0.5	0.8	5C	0.4	0.3	0.9
2D	0.4	0.2	0.8	5D	0.4	0.4	0.9
2E	0.6	0.2	0.8	5E	0.5	0.2	0.9
2F	0.4	0.3	0.8	5F	0.4	0.2	0.9
3A	0.3	0.2	0.8	6A	0.4	0.2	1.0
3B	0.3	0.2	0.8	6B	0.5	0.3	1.0
3C	0.4	0.2	0.8	6C	0.5	0.3	0.9
3D	0.4	0.2	0.9	6D	0.4	0.3	0.9
3E	0.4	0.3	0.9	6E	0.4	0.4	1.0
3F	0.5	0.2	0.9	6F	0.4	0.3	1.1

**Table 2:** Uncertainty calibration cube results.

U_X,cube_ (k_cube_ = 2) (µm)	U_Y,cube_ (k_cube_ = 2) (µm)	U_Z,cube_ (k_cube_ = 2) (µm)
0.6	0.5	1.1

**Table 3:** Experimental repeatability and systematic error (bias) results for the probe.

				X-Axis	Y-Axis	Z-Axis
Scanning probe	Repeatability	$S_{c,global}$	(µm)	2.0	2.0	2.1
	Bias range	$\Delta X_{global}$	(µm)	±4.9	±5.2	±5.8

**Table 4:** Local uncertainty results of the analog tactile probe calibration.

Sphere Cube Position	U_X_ (k = 2, n_i_ = 1) (µm)	U_Y_ (k = 2, n_i_ = 1) (µm)	U_Z_ (k = 2, n_i_ = 1) (µm)	Sphere Cube Position	U_X_ (k = 2, n_i_ = 1) (µm)	U_Y_ (k = 2, n_i_ = 1) (µm)	U_Z_ (k = 2, n_i_ = 1) (µm)
1A	1.9	4.1	1.5	4A	2.6	1.9	4.3
1B	2.0	3.8	1.3	4B	5.4	1.4	1.5
1C	2.0	3.0	1.1	4C	2.8	1.5	6.0
1D	2.4	3.3	4.8	4D	5.7	6.1	2.2
1E	1.6	2.9	2.1	4E	4.0	3.4	5.7
1F	2.6	4.6	5.5	4F	1.1	2.9	2.7
2A	0.7	1.9	0.8	5A	3.6	6.3	1.9
2B	1.8	1.4	1.3	5B	4.9	2.5	2.4
2C	1.4	3.1	4.2	5C	6.7	7.0	1.7
2D	2.6	3.6	5.7	5D	3.8	4.1	3.0
2E	1.3	3.8	6.0	5E	2.5	3.9	7.0
2F	0,9	2.8	3.5	5F	3.7	5.1	6.2
3A	1.8	5.2	7.2	6A	3.2	3.9	7.4
3B	1.6	3.2	5.3	6B	3.5	3.6	8.0
3C	1.2	3.3	7.9	6C	6.1	6.0	7.8
3D	0.7	1.3	2.8	6D	4.6	6.6	1.3
3E	1.3	2.0	2.1	6E	5.4	2.5	3.5
3F	5.4	3.8	4.0	6F	5.3	6.9	8.0

**Table 5:** Global uncertainty results of the analog tactile probe calibration.

U_X_ (k = 2, n_i_ = 1) (µm)	U_Y_ (k = 2, n_i_ = 1) (µm)	U_Z_ (k = 2, n_i_ = 1) (µm)
6.7	7.0	8.0

## Data Availability

The raw data supporting the conclusions of this article will be made available by the authors upon request.
